# Desoxyrhapontigenin up-regulates Nrf2-mediated heme oxygenase-1 expression in macrophages and inflammatory lung injury

**DOI:** 10.1016/j.redox.2014.02.001

**Published:** 2014-02-18

**Authors:** Ran Joo Choi, Mao-sheng Cheng, Yeong Shik Kim

**Affiliations:** a Natural Products Research Institute, College of Pharmacy, Seoul National University, Seoul 151-742, Korea; b School of Pharmaceutical Engineering, Shenyang Pharmaceutical University, Shenyang 110016, PR China

**Keywords:** Desoxyrhapontigenin, Nrf2, HO-1, Macrophages, Acute lung injury

## Abstract

Heme oxygenase-1 (HO-1) is an important anti-inflammatory, antioxidative and cytoprotective enzyme that is regulated by the activation of the major transcription factor, nuclear factor (erythroid-derived 2)-like 2 (Nrf2). In the present study, six stilbene derivatives isolated from *Rheum undulatum* L. were assessed for their antioxidative potential. In the *tert*-butylhydroperoxide (*t*-BHP)-induced RAW 264.7 macrophage cell line, desoxyrhapontigenin was the most potent component that reduced intracellular reactive oxygen species (ROS) and peroxynitrite. In response to desoxyrhapontigenin, the mRNA expression levels of antioxidant enzymes were up-regulated. An electrophoretic mobility shift assay (EMSA) confirmed that desoxyrhapontigenin promoted the DNA binding of Nrf2 and increased the expression of antioxidant proteins and enzymes regulated by Nrf2. Further investigation utilizing specific inhibitors of Akt, p38, JNK and ERK demonstrated that the phosphatidylinositol 3-kinase (PI3K)/Akt pathway mediates HO-1 expression. Moreover, the increase in Nrf2 expression mediated by treatment with desoxyrhapontigenin was reversed by Nrf2 or Akt gene knock-down. In the LPS-induced *in vivo* lung inflammation model, pretreatment with desoxyrhapontigenin markedly ameliorated LPS-induced lung inflammation and histological changes. Immunohistochemical analysis of Nrf2, HO-1 and p65 was conducted and confirmed that treatment with desoxyrhapontigenin induced Nrf2 and HO-1 expression but reduced p65 expression. These findings suggest that desoxyrhapontigenin may be a potential therapeutic candidate as an antioxidant or an anti-inflammatory agent.

## Highlights

• Enhancement of the levels of antioxidant enzymes by desoxyrhapontigenin.
• Promotion of DNA binding affinity of Nrf2 in RAW 264.7 macrophages.
• Induction of HO-1 expression and inhibition of Keap1 by desoxyrhapontigenin via the Akt pathway.
• Amelioration of LPS-induced inflammatory lung injury in mice.

## Graphical abstract

A proposed pathway for desoxyrhapontigenin-induced Nrf/ARE-mediated up-regulation of antioxidant genes via the activation of Nrf2 signaling.

**Figure:**
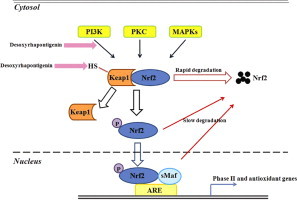

## Introduction

Nuclear factor (erythroid-derived 2)-like 2 (Nrf2) is a transcription factor that is encoded by the NFE2L2 gene in humans [1]. The function of Nrf2 and its downstream target antioxidant genes have been shown to be important for cytoprotection against reactive oxygen species (ROS), reactive nitrogen species (RNS) and cellular damage [2]. Nrf2 is constitutively located in the cytoplasm and bound to Kelch-like ECH-associated protein 1 (Keap1), also known as the Nrf2 repressor. The activation of Nrf2 is primarily controlled by Keap1, which constantly ubiquitylates Nrf2 and leads to its degradation in the cytoplasm. In response to oxidative stress, Nrf2 is released from Keap1, translocates into the nucleus, forms a heterodimer with the small Maf protein and binds to antioxidant-related elements (ARE) in the promoter region of phase II detoxifying enzymes and cytoprotective genes [3]. These enzymes include NAD(P)H quinone oxidoreductase (NQO1), glutathione *S*-transferase (GST), heme oxygenase-1 (HO-1), glutathione peroxidase (GPx), glutamate cysteine ligase (GCL) and peroxiredoxin I (Prx I) [4].

HO-1 is the major anti-inflammatory and cytoprotective enzyme among the genes that are regulated by Nrf2 activation [5]. HO is an enzyme that catalyzes the degradation of heme to produce biliverdin, iron and carbon monoxide (CO). The various biochemical actions of heme degradation products and their metabolic derivatives contribute to the cytoprotective functions of HO-1. Three isoforms of heme oxygenase exist but only HO-1 is induced in response to stress. HO-1 expression is up-regulated in response to various forms of inflammatory stimuli, and this up-regulation is associated with its anti-inflammatory actions. HO-1 has critical roles in both biosynthesis and degradation pathways of heme. When HO-1 catalyzes heme to biliverdin and bilirubin, these degradation products have antioxidant properties. Iron, which is degraded from heme, may also contribute to cytoprotective properties against oxidative stress or inflammation by the stimulation of ferritin synthesis followed by iron detoxification [6], [7]. Mice with HO-1 deficiency have a distinct phenotype with an increased inflammatory state, suggesting that HO-1 is critical for the resolution of inflammation [8].

The interaction between Nrf2 and NF-κB is interesting because numerous phytochemicals that have anti-inflammatory, antioxidative or anti-cancer properties suppress NF-κB signaling and activate the Nrf2 pathway [9]. Jin et al. reported that Nrf2-deficient mice with head injury showed a higher cerebral NF-κB activation than wild-type mice [10]. The mitogen-activated protein kinase (MAPK) family contributes to both the Nrf2 and NF-κB pathways. NF-κB competes with Nrf2 for binding to the transcriptional coactivator CREB-binding protein (CBP) and also promotes the binding of the corepressor histone deacetylase 3 (HDAC3) to ARE. Thus, NF-κB may be a negative regulator of the Nrf2 pathway [11].

This study investigated the biological activities of isolated components from the rhubarb plant. Because rhubarb is known to have anti-inflammatory properties, it has the potential to exhibit diverse biological activities. Stilbene compounds are primarily present in grapes and red wine, but *Rheum undulatum* L. is also known to contain a number of stilbene derivatives. Moreover, resveratrol is a well-known phytochemical that possesses various biological properties, including anticancer, antiviral, anti-inflammatory and antioxidant activities [12], [13], [14]. Its extract and fractions showed apoptotic effect on oral cancer cells and killed bacteria which are involved in dental diseases [15], [16], [17]. Furthermore, *R. undulatum* showed potent inhibitory effects on diabetes and obesity due to its major components anthraquinones as well as stilbene derivatives [18], [19]. Pharmacokinetic study of major compounds present in this plant has been also carried out [20], [21]. Anti-inflammatory potentials of stilbene components isolated from *R. undulatum* have been recently studied and published [22]. However, *R. undulatum* L. cannot be used as an herbal medicine in Korea, because it is not listed in the Korean Pharmacopeia and does not contain sennoside A which is a marker component. However, the use of *R. undulatum* L. needs to be encouraged because, although it lacks sennoside A, it has sufficient amounts of anthraquinones as well as stilbenes. Several research groups studying stilbene analogs found that stilbenes are valuable chemicals [23], [24], [25]. Thus, the presence of the stilbene derivatives in *R. undulatum* L. is distinctly noteworthy. In this report, six stilbene derivatives isolated from *R. undulatum* L. were studied including resveratrol, a well-known bioactive compound and others with similar structures.

## Material and methods

### Plant material

The cultivated rhubarb rhizome was purchased at a local herbal market in Daejeon, Korea in July 2006 and was identified by Professor KiHwan Bae. A voucher specimen (CNU-1345) was deposited in the herbarium of College of Pharmacy at Chungnam National University in Korea. Furthermore, the chemical-based species classification has been conducted to identify rhubarb species [26].

### Isolation of stilbene derivatives

**Fig. 1:**
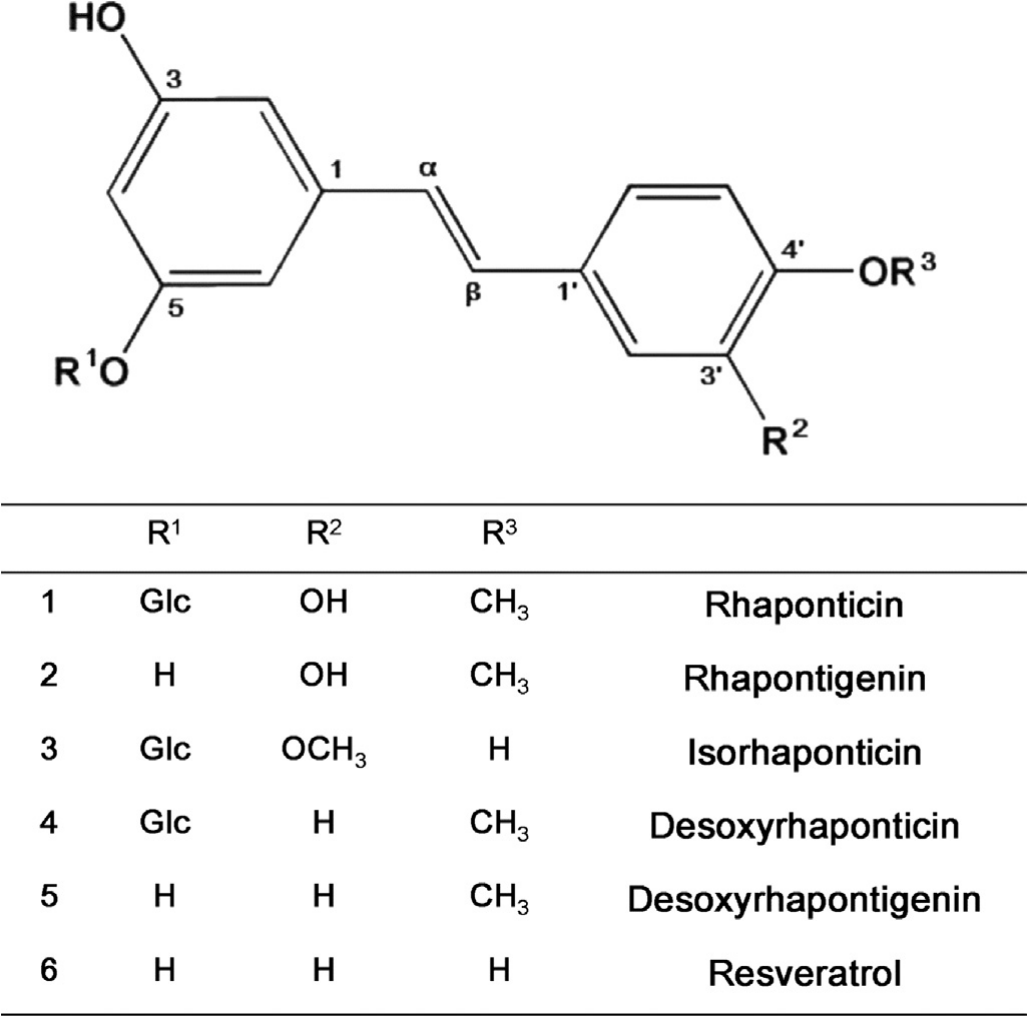
The structures of the stilbene derivatives that were assessed in this study, Rhaponticin (1), rhapontigenin (2), isorhaponticin (3), desoxyrhaponticin (4), desoxyrhapontigenin (5) and resveratrol (6) were studied.

The dried and milled rhizomes of rhubarb (4.7 kg) were extracted three times with 20 L of 70% ethanol. The ethanol extracts were combined and concentrated, and the residue (650 g) was used to isolate rhaponticin (**1**), rhapontigenin (**2**), isorhaponticin (**3**), desoxyrhaponticin (**4**), desoxyrhapontigenin (**5**), and resveratrol (**6**) as described in a previous report [27]. The structures of the stilbene derivatives are shown in Fig. 1.

### Cell culture

The RAW 264.7 murine macrophages were obtained from the American Type Culture Collection (Manassas, VA, USA). These cells were maintained at sub-confluence in a 95% air and 5% CO_2_-humidified atmosphere at 37 °C. DMEM supplemented with 10% fetal bovine serum (FBS), 100 U/mL of penicillin and 100 μg/mL of streptomycin was used for routine subculturing and *in vitro* experiments (GenDEPOT, Barker, TX, USA). Unless otherwise indicated, all of the chemicals that were used in the current study were purchased from Sigma-Aldrich Co. (St. Louis, MO, USA). All of the samples were dissolved in dimethyl sulfoxide (DMSO), and the final DMSO concentrations were <0.1%.

### Cell viability

The measurements of cell viability after exposure to the stilbene derivatives were performed using an MTT ((4,5-dimethylthiazol-2-yl)-2,5-diphenyl tetrazolium bromide) assay. Briefly, RAW 264.7 cells were plated at a density of 1×10^4^ cells per well in a 96-well plate and incubated at 37 °C for 24 h. The cells were treated with various concentrations of each stilbene compound or the vehicle alone and incubated at 37 °C for an additional 24 h. After the incubation for 24 h, the medium was removed, and 100 μL of MTT (0.5 mg/mL in DPBS) solution was added into each well, and the cells were incubated in the same conditions for an additional 2 h. MTT is cleaved by living cells into visible formazan crystals during this incubation. The formazan crystals were then solubilized in DMSO, and the resulting absorbance was measured at 595 nm using an enzyme-linked immunosorbent assay (Molecular Devices, Sunnyvale, CA, USA). The relative cell viability was calculated and compared with the absorbance of the untreated control group.

### Intracellular ROS and peroxynitrite

The intracellular ROS scavenging activity of silbene derivatives was measured using the oxidant-sensitive fluorescent probe, DCFH-DA [28]. DCFH-DA is converted to DCFH by deacetylases within the cells and is oxidized by a variety of intracellular ROS to the fluorescent compound DCF. RAW 264.7 cells were seeded in a black 96-well plate. After 24 h, the cells were treated with the indicated concentrations of stilbenes for 2 h and were incubated for 1 h with *t*-BHP (200 μM) to induce ROS generation. After the cells were incubated with DCFH-DA (20 μM) for 30 min, the fluorescence intensity was scanned by a 96-well microplate fluorometer (Molecular Devices Gemini XS, Sunnyvale, CA, USA) at an excitation wavelength of 485 nm and an emission wavelength of 538 nm.

Peroxynitrite scavenging was measured by monitoring the oxidation of dihydrorhodamine 123 (DHR 123) as described in a previous report with some modifications [29]. Briefly, RAW 264.7 cells were seeded in a black 96-well plate and incubated for 24 h. The indicated concentrations of the stilbene derivatives were added, and the cells were incubated for 2 h. Subsequently *t*-BHP (200 μM) was added to induce peroxynitrite generation, and the cells were further incubated for 1 h. Rhodamine solution (50 mM sodium phosphate buffer, 90 mM sodium chloride, 5 mM potassium chloride, 5 mM diethylenetriaminepentaacetic acid (DTPA) and DHR 123) was then added and incubated with the cells for another 1 h. The final fluorescence intensity was measured on a microplate fluorometer (Molecular Devices Gemini XS, Sunnyvale, CA, USA) with excitation and emission wavelengths of 485 nm and 538 nm, respectively.

### Western immunoblot analysis

Nuclear and cytoplasmic protein extracts from RAW 264.7 cells were prepared after treatment with 50 μM of desoxyrhapontigenin for the indicated time periods. Total protein extracts from the specified time periods were also prepared for the detection of Keap1, an inhibitor of Nrf2. To elucidate the upstream signaling pathway involved in desoxyrhapontigenin-mediated Nrf2 activation and HO-1 induction, specific inhibitors of PI3K/Akt (LY294002, BioVision, Milpitas, CA, USA), p38 (SB202190), JNK (SP600125) and ERK (U0126) were used. Protein extracts were separated by 10% SDS-PAGE, electro-transferred to nitrocellulose membranes (Whatman GmbH, Dassel, Germany), blotted with each primary antibody (1:1000) and the corresponding secondary antibody (1:5000), and detected with the WEST-ZOL^®^ (plus) Western Detection System (iNtRON, Korea). The target bands were quantified using the UN-SCAN-IT™ gel 6.1 software (Silk Scientific Corp, Orem, UT, USA).

### Quantitative real-time reverse transcriptase polymerase chain reaction

Total RNA (1 μg) was converted to cDNA by PCR (Genius FGEN05TD, Teche, England) using the iScript^TM^ cDNA Synthesis Kit (BIO-RAD, Hercules, CA, USA) under the following conditions: 25 °C for 5 min, 42 °C for 30 min and 85 °C for 5 min. Quantitative real-time reverse-transcription polymerase chain reaction (RT-PCR) analysis was performed using the Applied Biosystems 7300 real-time PCR system and software (Applied Biosystems, Carlsbad, CA, USA). Quantitative PCR was conducted in 0.2 mL PCR tubes with forward and reverse primers and the SYBR green working solution (iTaq™ Universal SYBR Green Supermix, BIO-RAD, Hercules, CA, USA), using a custom PCR master mix with the following conditions: 95 °C for 30 s, followed by 40 cycles of 95 °C for 15 s, 55, 58 or 68 °C for 20 s and 72 °C for 35 s (amplification temperature for each gene is indicated in Table 1). The melting point, optimal conditions and the specificity of the reaction were first determined. The sequences of the PCR primers were previously described [30], [31], [32]. The copy number of each transcript was calculated as the relative copy number normalized to the β-actin copy number.

**Table 1:** The sequences of the PCR primers and amplification temperature.

Genes	Forward primer	Reverse primer	˚C
β-actin	CCCACTCCTAAGAGGAGGATG	AGGGAGACCAAAGCCTTCAT	55
HO-1	CACGCATATACCCGCTACCT	CCAGAGTGTTCATTCGAGA	58
SOD2	GCGGTCGTGTAAACCTCAT	CCAGAGCCTCGTGGTACTTC	58
SOD3	CTGAGGACTTCCCAGTGAC	GGTGAGGGTGTCAGAGTGT	58
NQO1	TTCTCTGGCCGATTCAGAGT	GGCTGCTTGGAGCAAAATG	58
GR	CAACACAGTGGCCATTCAC	TTGTTTCTCATGGACCACCA	58
GCLS	TGGAGCAGCTGTATCAGTG	AGAGCAGTTCTTTCGGGT	58
Prx-1	CACTGACAAACATGGGGAAGT	TTTGCTCTTTTGGACATCAGG	68

### Electrophoretic mobility shift assay

To assess the activation of Nrf2, an electrophoretic mobility shift assay (EMSA) was performed as previously described with some modifications [33]. RAW 264.7 cells were seeded at a density of 1×10^6^ cells per well in 6-well plates and incubated at 37 °C for 24 h. Subsequently, the cells were incubated with desoxyrhapontigenin for the indicated time points. The nuclear extracts prepared from the cells were incubated with a ^32^P-end-labeled 22-mer double-stranded Nrf2 consensus oligonucleotide (Santa Cruz Biotechnology Inc, Santa Cruz, CA, USA: sequences: 5׳-TGG GGA ACC TGT GCT GAG TCA CTG GAG-3׳ and 3׳-ACC CCT TGG ACA CGA CTC AGT GAC CTC-5׳) for 30 min at room temperature, and the DNA-protein complexes were separated from the free oligonucleotides on 6% native polyacrylamide gels. The gels were dried and visualized using a BAS-1500 apparatus (Fuji, Tokyo, Japan).

### Transient transfection of small RNA interference (siRNA)

RAW 264.7 cells were seeded at a density of 3×10^5^ cells/well in 6-well plates and allowed to reach approximately 50% confluence on the day of transfection. The cells were transfected with 50 nM of scrambled siRNA, Nrf2 siRNA or Akt siRNA using Lipofectamine RNAiMAX (Invitrogen, Life technologies, CA, USA). After 4 h incubation, the medium was replaced with fresh medium with FBS.

### Production of LPS-treated mice

Five ICR male mice per each group were used for the acute lung inflammation model according to previous report with some modifications [34]. Desoxyrhapontigenin (2.5 and 10 mg/kg) was intraperitoneally administered to mice every other day for one week. On the seventh day, 5 mg/kg of LPS (*E. coli*-026:B6, Sigma) was injected intraperitoneally. Subsequently, the mice were sacrificed after 24 h of monitoring, and the lung tissues were excised. The lung tissues were fixed in 10% formalin, dehydrated with a gradient of ethanol and embedded in paraffin. Tissue sections (4 μm) were then dewaxed and rehydrated according to a standard protocol. For histologic analysis, the sections were stained with hematoxylin and eosin (H&E). For immunohistochemical analysis of Nrf2, HO-1 and p65, the sections were treated with 1% hydrogen peroxide for 10 min and rinsed with PBS after deparaffinization. Then, sections were blocked with 2% normal blocking serum in PBS at room temperature for 60 min, followed by incubation with Nrf2, HO-1 or p65 antibodies. Sections were analyzed using an inverted microscope (Olympus CKX41, Japan). Then, the sections were further analyzed and quantified by the ImageJ version1.46r software (Wayne Rasband, NIH, USA).

### Molecular docking study

The molecular docking study was performed with LigandFit (Accelrys Inc., CA, USA), which uses a shape-based method for accurately predicting binding poses within the receptor active site. The crystal structure of human KEAP1 (PDB code: 1ZGK) was used in the molecular modeling study. The candidate poses were minimized in the context of the active site using a grid-based method for evaluating protein–ligand interaction energies. The simulation results were analyzed with Discovery Studio (Accelrys Inc., CA, USA) [35].

### Data analysis

The results are expressed as the means±standard deviation (S.D.). Analysis of variance (ANOVA) with Dunnett׳s *t*-test was used for the statistical analysis of multiple comparisons. *P*-values less than 0.05 were considered statistically significant. [^⁎^*P*<0.05; ^⁎⁎^*P*<0.01; ^⁎⁎⁎^*P*<0.001].

## Results

### Cell viability

**Fig. 2:**
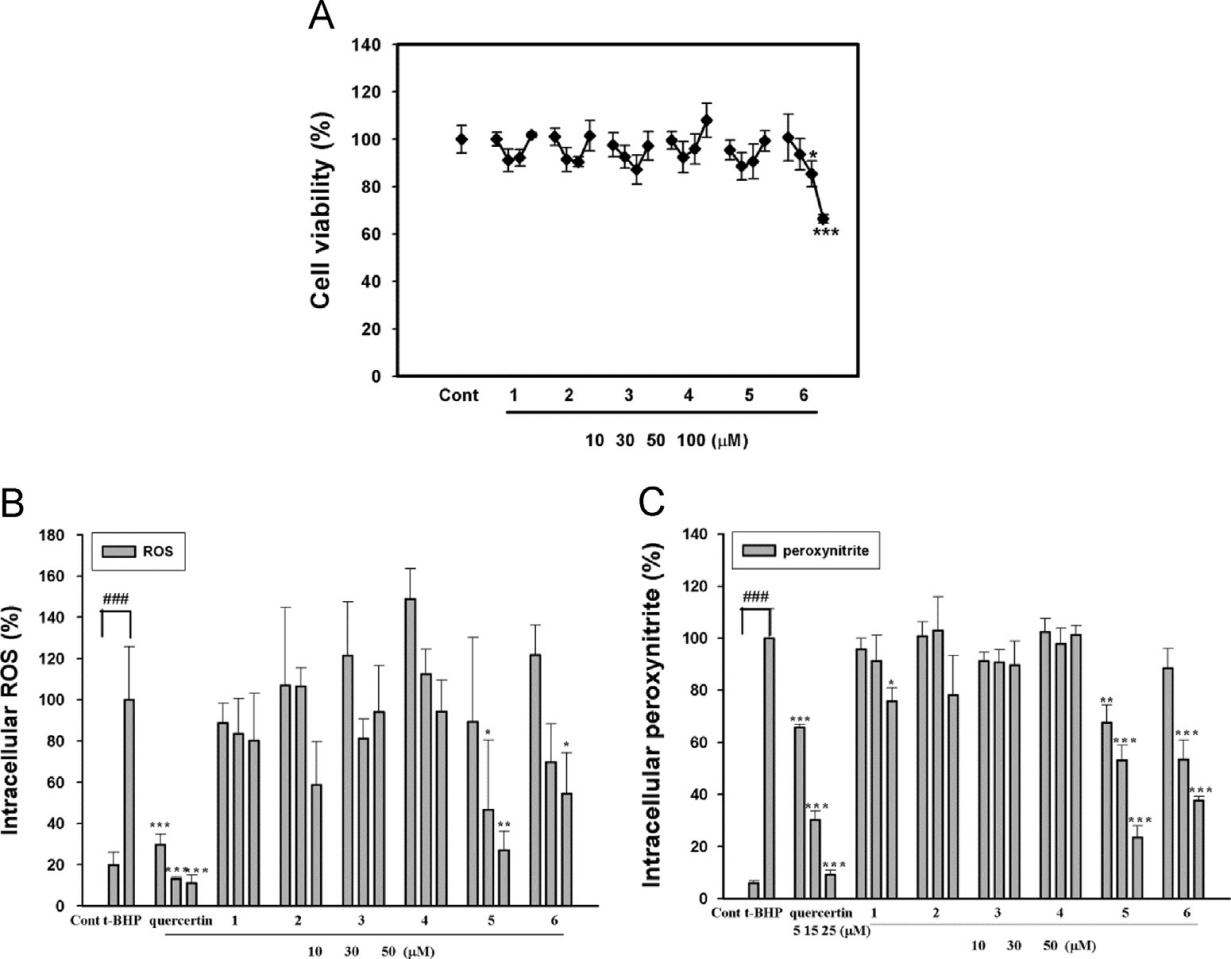
Effects of stilbene derivatives on intracellular ROS and peroxynitrite, The effects of stilbene compounds on *t*-BHP-induced ROS (A) and peroxynitrite (B) generation in RAW 264.7 cells were evaluated. Desoxyrhapontigenin and resveratrol had dose-dependent inhibitory effects on *t*-BHP-induced ROS and peroxynitrite levels. The data were obtained from three independent experiments and are expressed as the means±S.D. In this figure, (⁎), (⁎⁎) and (⁎⁎⁎) indicate a significant difference from the *t*-BHP-induced group at the *P*<0.05, *P*<0.01 and *P*<0.001 levels, respectively, whereas (###) indicates a significant difference from the unstimulated control group at the *P*<0.01 level. Quercetin was used as a positive control.

To evaluate cell viability during the experimental treatments in this study, an MTT assay was conducted under the conditions that were identical to those used for the treatments. The cell viability percentages of RAW 264.7 macrophages are shown in Fig. 2A. In viable cells, mitochondrial succinate dehydrogenase converts MTT into visible formazan crystals that can be measured by O.D. values at 595 nm. Incubation with 10, 30, 50 or 100 µM of five stilbene derivatives (**1**–**5**) for 24 h showed no significant change in cell viability compared to the vehicle-treated cells (Cont). However, treatment with resveratrol produced significant differences in cell viability at doses of 50 and 100 µM.

### Effects of stilbene derivatives on intracellular ROS and peroxynitrite

The effects of stilbene compounds on *t*-BHP-induced ROS and peroxynitrite generation in RAW 264.7 cells were evaluated (Fig. 2B and C). Incubation with *t*-BHP elevated intracellular ROS and peroxynitrite levels that were represented by fluorescence intensity. Desoxyrhapontigenin and resveratrol displayed dose-dependent inhibitory effects on *t*-BHP-induced ROS and peroxynitrite levels. The half maximal inhibitory concentration (IC_50_) values of desoxyrhapontgenin against ROS and peroxynitrite were 32.83 μM and 28.22 μM, respectively. Resvereatrol showed IC_50_ values of 49.07 μM for ROS and 37.82 μM for peroxynitrite in *t*-BHP-induced RAW 264.7 macrophages.

### Effects of desoxyrhapontigenin on the mRNA expression of antioxidant enzymes and the protein expression of HO-1

**Fig. 3:**
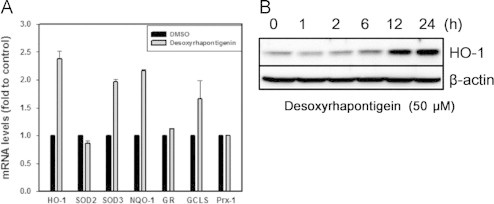
Effects of desoxyrhapontigenin on the mRNA expression of antioxidant enzymes and the protein expression of HO-1, RAW 264.7 cells were treated with 50 μM desoxyrhapontigenin. Reverse transcription and real-time PCR analyses are described in “Section 2”. The relative phase II enzyme expression levels (in terms of 2^−ΔCt^) were determined by real-time PCR and calculated by subtracting the *C*_*t*_ value for β-actin from the *C*_*t*_ values for the phase II enzymes (Δ*C*_*t*_=*C*_*t*(antioxidant)_−*C*_*t*(β-actin)_) (A). Western blot analysis was conducted to confirm the time-course of HO-1 protein expression induction by desoxyrhapontigenin. The cells were treated with desoxyrhapontigenin at 50 μM for the indicated time periods, and the total proteins were extracted to detect the expression of HO-1 (B).

Real-time PCR analysis was performed to determine which of the phase II enzymes are induced by desoxyrhapontigenin. Activation of the antioxidant response element (ARE) by the Nrf2 pathway causes the induction of the phase II enzymes (e.g., HO-1, NQO1, GR, SOD, GCLS and Prx-1). The mRNAs for HO-1, superoxide dismutase 3 (SOD3), NQO1 and γ-glutamate cysteine ligase subunit (GCLS) were increased in RAW 264.7 cells treated with desoxyrhapontigenin, whereas SOD2, glutathione reductase (GR) and Prx-1 mRNA levels were unchanged (Fig. 3A). Western blot analysis was conducted to confirm the induction of HO-1 protein expression by desoxyrhapontigenin (Fig. 3B).

### Desoxyrhapontigenin increased the levels of the Nrf2 transcription factor and reduced the expression of Keap1

**Fig. 4:**
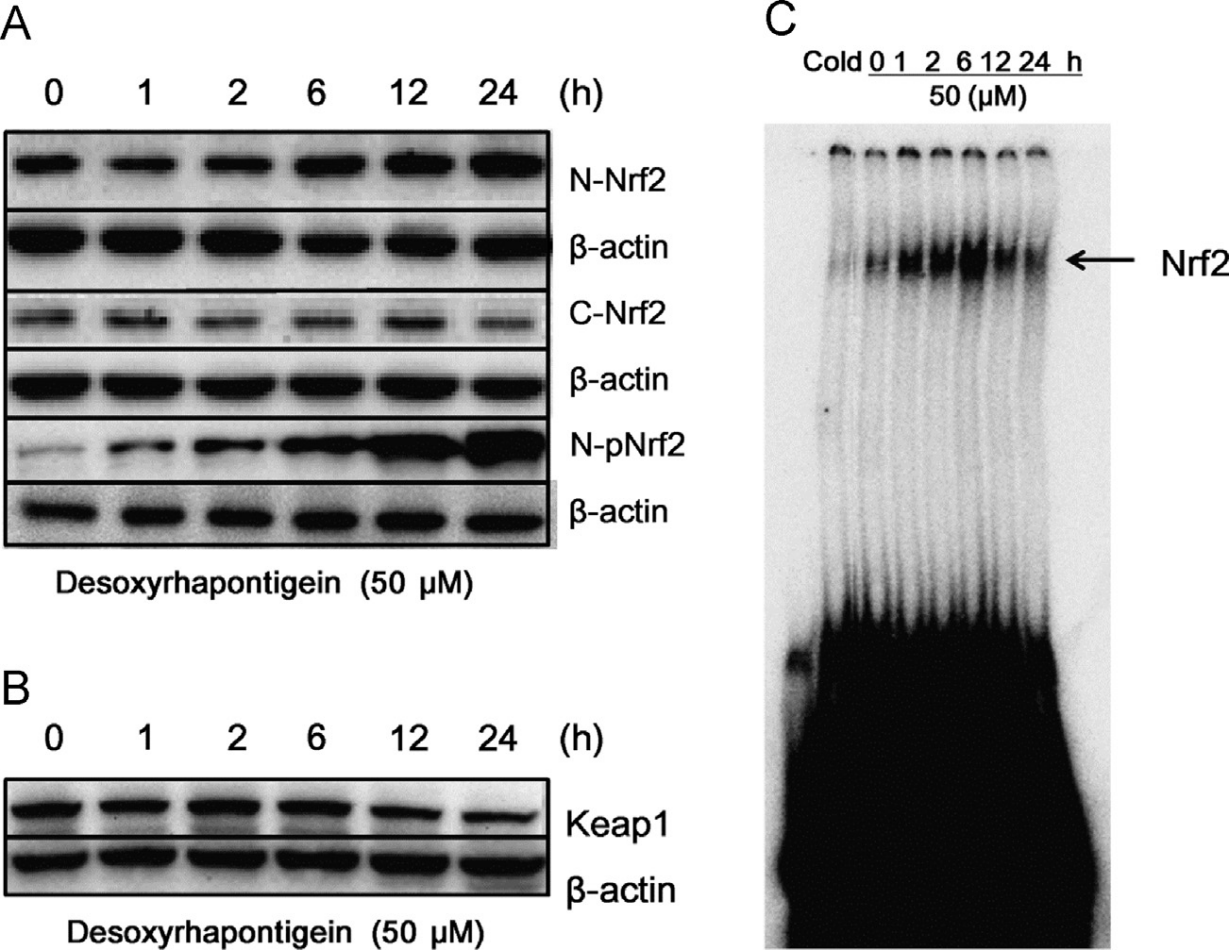
Effects of desoxyrhapontigenin on the protein expression levels of Nrf2, p-Nrf2 and Keap1 and the transcriptional activity of the ARE in RAW 264.7 macrophages, Nuclear and cytoplasmic extracts from RAW 264.7 cells were prepared after treatment with desoxyrhapontigenin at 50 μM for the indicated time periods (A). Total protein extracts from the specified time periods were also prepared for detecting Keap1, an inhibitor of Nrf2 (B). To confirm the specific DNA–protein interaction, an EMSA was performed with excess amounts of unlabeled Nrf2 oligonucleotide in a competition assay (Cold) (C).

Most of the genes encoding phase II detoxifying and antioxidant enzymes have an ARE sequence in their promoter regions. Nrf2 is an important transcription factor that regulates the ARE-driven expression of proteins such as HO-1 and other phase II enzymes. Desoxyrhapontigenin treatment increased both phosphorylated Nrf2 and Nrf2 accumulation in the nuclear fraction (Fig. 4A). Moreover, desoxyrhapontigenin down-regulated the expression of the Keap1 protein, an inhibitor of Nrf2, in a time-dependent manner (Fig. 4B). To determine the role of Nrf2 in the transcriptional activation of ARE, an EMSA was performed. Desoxyrhapontigenin increased Nrf2 binding to the ARE sequence (Fig. 4C).

### Induction of HO-1 and activation of Nrf2 by desoxyrhapontigenin via the PI3K/Akt pathway

**Fig. 5:**
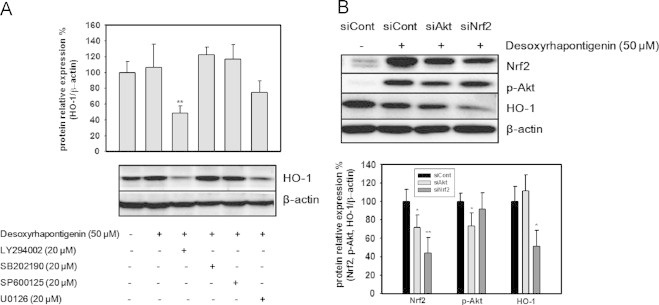
Effects of specific inhibitors on the induction of HO-1 by desoxyrhapontigenin and the effects of siAkt and siNrf2 on the induction of Nrf2 and HO-1 by desoxyrhapontigenin, To elucidate the upstream signaling pathway involved in desoxyrhapontigenin-mediated Nrf2 activation and HO-1 induction, specific inhibitors of PI3K/Akt (LY294002), p38 (SB202190), JNK (SP600125) and ERK (U0126) were used. Inhibition of the Akt pathway reduced the capacity of desoxyrhapontigenin to induce HO-1 protein (A). To confirm these findings, cells were transfected with siNrf2 or siAkt and treated with desoxyrhapontigenin. Activation of Nrf2 by desoxyrhapontigenin was inhibited in siAkt or siNrf-transfected cells, and HO-1 induction was inhibited in siNrf2-transfected cells (B). The results of three experiments are expressed as the means±S.D. Significant differences from the desoxyrhapotigenin only-treated group are shown by (⁎) and (⁎⁎) which indicate the *P*<0.05 and *P*<0.01 levels, respectively.

To elucidate the upstream signaling pathway involved in desoxyrhapontigenin-mediated Nrf2 activation and HO-1 induction, specific inhibitors of PI3K/Akt (LY294002), p38 (SB202190), JNK (SP600125) and ERK (U0126) were used. Inhibition of the Akt pathway reduced the capacity of desoxyrhapontigenin to induce increased HO-1 protein levels (Fig. 5A). To confirm these results, cells were transfected with siNrf2 or siAkt and treated with desoxyrhapontigenin. Activation of Nrf2 by desoxyrhapontigenin was inhibited in siAkt or siNrf-transfected cells, and HO-1 induction was inhibited in siNrf2-transfected cells (Fig. 5B).

### Effects of desoxyrhapontigenin in a mouse model of LPS-induced inflammatory lung injury

**Fig. 6:**
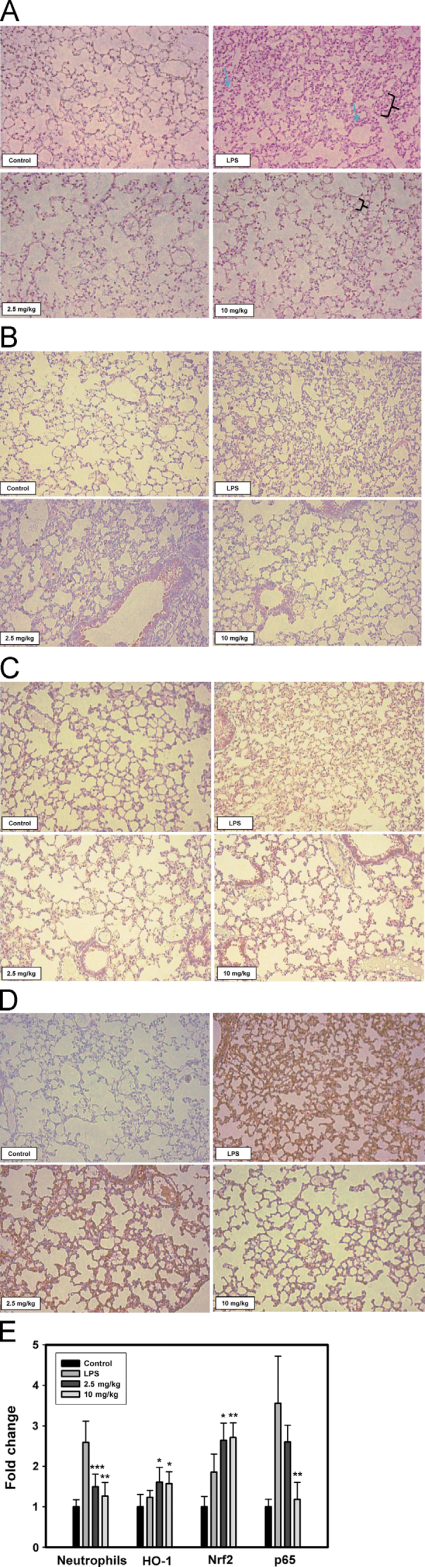
Histological changes of desoxyrhapontigenin treatment in LPS-induced lung inflammation and immunohistochemistry analysis of Nrf2, HO-1 and p65, Lung tissues were collected at 24 h after injection of LPS and subjected to H and E staining and immunohistochemistry (×200). The alveolar septal wall was thickened (a brace) and destroyed by prominent neutrophil infiltration (arrows) and alveolar hemorrhage in the LPS-treated group. Desoxyrhapontigenin treatment reduced neutrophil infiltration and alveolar hemorrhage, whereas the thickened alveolar septal wall returned to normal (A). Photomicrographs of the HO-1, Nrf2 and p65-immunostained lung sections confirmed that desoxyrhapontigenin induces HO-1 (B) and Nrf2 (C), but reduces p65 (D) in LPS-treated lung tissues (brown dots). The sections were quantified by ImageJ version1.46r software (E). The results are expressed as the means±S.D. Significant differences from the LPS-treated group are shown by (⁎), (⁎⁎) and (⁎⁎) which indicate the *P*<0.05, *P*<0.01 and *P*<0.001 levels, respectively.

Lung tissues were collected 24 h after injection with LPS and subjected to H and E staining and immunohistochemistry (Fig. 6). The inflammation was reduced by desoxyrhapontigenin treatment as visualized by H and E staining. The thickened alveolar septal wall induced by LPS was reduced in the desoxyrhapontigenin-treated groups. Furthermore, desoxyrhapontigenin reduced neutrophil infiltration and alveolar hemorrhage (Fig. 6A). Immunohistochemical analysis of Nrf2 and HO-1 was performed to confirm that treatment with desoxyrhapontigenin induced Nrf2 (Fig. 6C) and HO-1 (Fig. 6B) expression. The Nrf2 or HO-1 protein was distributed in the desoxyrhapontigenin-treated lung tissues, stained as brown granules or dots. In contrast, in the NF-κBp65-immunostained lung sections, desoxyrhapontigenin suppressed p65 expression when compared with the LPS-treated group (Fig. 6D).

### Molecular docking study

LigandFit employs a shape comparison filter and Monte Carlo conformational analysis for generating poses consistent with the active site shape. The binding affinities were calculated with the LigScore scoring function, yielding a DOCK_SCORE value of 50.715 for desoxyrhapontigenin. However, LigandFit could not match resveratrol to the site or produce any poses. Desoxyrhapontigenin forms hydrogen bonds with Arg415, Val418, Cys368 and Val606.

## Discussion

Oxidative stress is defined as an imbalance between the production of free radicals and their elimination by cellular defense mechanisms. ROS are chemically reactive molecules containing oxygen and are produced by normal cellular metabolism. Generated ROS in mitochondria and cells cause damage to lipids, proteins and DNA [36]. To protect against produced ROS, such as superoxide anion (O_2_^−^^•^), hydrogen peroxide (H_2_O_2_), hydroxyl radical (OH^•^) and organic peroxides, cellular antioxidant enzymes including superoxide dismutase, catalase and glutathione peroxidase are induced. In addition to ROS, reactive nitrogen species (RNS) are molecules derived from nitric oxide (^•^NO) and superoxide (O_2_^−^^•^). Neither NO nor O_2_^−^^•^ is toxic *in vivo* but powerful oxidant peroxynitrite (ONOO–) is formed by the reaction of ^•^NO and O_2_^−^^•^. Excessive production of peroxynitrite causes tissue damage or cell death and is involved in the pathophysiology of cardiovascular diseases, neurodegenerative disorders, local inflammation, diabetes and cancer [37].

Nrf2 is a transcription factor that induces antioxidant and phase II detoxifying enzymes that combat ROS or RNS. The promoters of cytoprotective proteins contain a specific consensus sequence called the ARE to which Nrf2 binds, promoting the transcription of ARE-related genes. Recent studies have reported that Nrf2-related ARE signaling is involved in attenuating inflammation-associated diseases [6]. During the early phase of inflammation-mediated tissue damage, activation of Nrf2-ARE may inhibit the production or expression of pro-inflammatory mediators including cytokines, COX-2 and iNOS. It is likely that the cytoprotective function of genes targeted by Nrf2 may cooperatively regulate the innate immune response and also repress the induction of pro-inflammatory genes. Polyphenolic compounds have been studied with respect to their antioxidative effects and ability to scavenge free radicals [38]. Resveratrol, a polyphenolic stilbene compound, has been shown to possess antioxidative, anti-inflammatory and anticancer effects [12], [39], [40].

Inflammation and oxidative stress are closely related. The key transcription factors Nrf2, NF-κB and AP-1 are essential, because the induction of Nrf2 leads to the up-regulation of antioxidant proteins and enzymes, whereas the inhibition of NF-κB and AP-1 blocks inflammatory mediators [41]. Previous studies have demonstrated that resveratrol inhibits the activities of NF-κB and AP-1 and induces detoxification and antioxidant enzymes through the Nrf2 pathway [41]. Thus, the hypothesis has been proposed in the present study that other stilbene components may act similarly and exhibit anti-inflammatory and antioxidative properties.

In this study, six stilbene derivatives from *R. undulatum* L. were assessed for their antioxidative properties. As expected, desoxyrhapontigenin and resveratrol reduced stimulated ROS and peroxynitrite levels. However, resveratrol caused cytotoxic effects at higher concentrations in RAW 264.7 macrophages while desoxyrhapontigenin showed no significant cytotoxicity (Fig. 2). Because desoxyrhapontigenin exhibited promising inhibitory effects on excessive ROS and peroxynitrite levels in the *t*-BHP-induced RAW 264.7 macrophage cell line, real-time PCR analysis was conducted to determine which of the phase II enzymes are induced by desoxyrhapontigenin. The phase II enzymes (e.g., HO-1, NQO1, GR, SOD, GCLS and Prx-1) are induced by activation of the ARE by the Nrf2 pathway. Desoxyrhapontigenin up-regulated the mRNA expression of HO-1, SOD3, NQO1 and GCLS, which are all transcribed by increased binding to the ARE (Fig. 3). Indeed, desoxyrhapontigenin induced HO-1 expression by targeting the ARE sequence, and this finding was supported by Nrf2 accumulation in the nucleus (Fig. 4). The increase in ARE-driven gene expression through Nrf2 activation may be regulated by two mechanisms [41].

In the first possible mechanism, Nrf2 is phosphorylated through kinase signaling pathways and translocates to the nucleus to bind to the ARE. When the PI3K, PKC or MAPK signaling pathways are activated, phosphorylated Nrf2 can be liberated from Keap1. Signaling pathways that are involved in the activation of Nrf2-mediated gene expression can be elucidated by using specific kinase inhibitors. Thus, desoxyrhapontigenin-mediated HO-1 induction was inhibited by chemical inhibitors of kinases such as PI3K/Akt (LY294002), p38 (SB202190), JNK (SP600125) and ERK (U0126). These inhibitors block the phosphorylation of kinases accordingly, thus the PI3K/Akt, p38, JNK or ERK signaling pathway may be hampered. Treatment with LY294002 blocked desoxyrhapontigenin-induced HO-1 up-regulation, whereas the blockage of other signaling pathways was unrelated. It seems that the activation of the PI3K/Akt pathway is closely correlated with HO-1 expression. RNA interference of the Akt or Nrf2 genes supports the hypothesis that desoxyrhapontigenin-induced ARE-driven genes, particularly HO-1, are stimulated by Nrf2 activation, which is regulated by the activation of Akt signaling (Fig. 5).

**Fig. 7:**
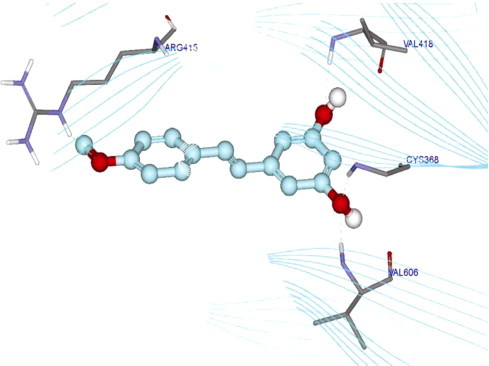
Binding modes of desoxyrhapontigenin within the protein active site of KEAP1 in the molecular docking study, The ligand is displayed in a ball-and-stick style in a cyan color, and the protein is displayed as line ribbon. Desoxyrhapontigenin forms hydrogen bonds with Arg 415, Val 418, Cys 368 and Val 606 of KEAP1.

Another possible mechanism of Nrf2 activation involves the cysteine residues of the Keap1 protein [42]. Keap1 has 25 cysteine residues, which make Keap1 an attractive target for thiol-reactive chemical species. Thus, inactivation of Keap1 is suggested to be another mechanism for Nrf2 activation. Modifying reactive cysteine residues by antioxidants abrogates the repressor activity of Keap1 and releases Nrf2 from the Keap1 complex. Sulforaphane is a potent natural product-derived Nrf2 activator that functions by modifying Keap1 cysteine residues. The C151, C38, C368 and C489 residues of Keap1 are the most readily modified by the phytochemical sulforaphane [43]. Molecular docking analysis was performed with desoxyrhapontigenin to assess its possible binding sites to Keap1 protein in this study. The results revealed that desoxyrhapontigenin forms hydrogen bonds with Arg 415, Val 418, Cys 368 and Val 606. This bonding could contribute to the activation of Nrf2 by desoxyrhapontigenin by inhibiting Keap1 repressor activity (Fig. 7).

In response to bacterial infection, the innate immune system is initiated for host defense. LPS-induced endotoxic shock or sepsis is characterized by severe tissue damage and the infiltration of neutrophils and macrophages into the lungs. During sepsis, Nrf2 is one of the most critical regulators that protect against inflammatory processes. Nrf2-deficient mouse models have been widely used to demonstrate the pivotal role of Nrf2 in LPS-induced pulmonary inflammation. NADPH oxidase-dependent ROS are closely involved in the pathophysiology of sepsis because disruption of the NADPH oxidase subunit gp91^phox^ in Nrf2-deficient mice significantly reduced LPS-induced systemic and lung inflammation [44]. Studies suggest that Nrf2-mediated cellular antioxidants are crucial modifiers of the pathogenesis of sepsis [45]. In addition to cecal ligation and puncture (CLP), which is a clinically relevant model of sepsis, challenge with LPS induced higher lethality in Nrf2-deficient mice than in wild-type mice. Moreover, sepsis and septic shock by induced LPS resulted in severe lung inflammation in Nrf2-deficient mice through the TLR4 signaling pathway. Because NF-κB is a major regulator of the TLR4 signaling pathway, NF-κB-mediated proinflammatory genes were up-regulated when Nrf2 was knocked out [46]. Thus, molecules that increase Nrf2 activity can be protective against the LPS-induced inflammatory response [47]. For instance, herbal medicines known as anti-inflammatory agents also show protective effects against LPS-induced lung inflammation through Nrf2 activation [48], [49].

Because desoxyrhapontigenin was investigated for its antioxidative properties in RAW 264.7 cells, the LPS-treated mouse model was used to further verify its effects. In preliminary studies, the proper doses of desoxyrhapontigenin were selected that could attenuate LPS-induced acute lung injury (data not shown). After *i.p.* administration of LPS resulted in macrophage infiltration, alveolar septal thickening and alveolar hemorrhage in lung tissues, administration of desoxyrhapontigenin significantly reduced these inflammation-mediated histological changes. Up-regulation of Nrf2 and HO-1 expression by desoxyrhapontigenin was also observed in lung tissue extracts, whereas p65 expression was reduced. Therefore, the effects of desoxyrhapontigenin were due to the induction of Nrf2-mediated HO-1 but inhibition of NF-κB.

## Conclusions

In the *t-*BHP-induced RAW 264.7 macrophage cell line, desoxyrhapontigenin decreased ROS and peroxynitrite production. Desoxyrhapontigenin itself induced phase II detoxifying genes, particularly HO-1. The induction of HO-1 by desoxyrhapontigenin was due to the activation of Nrf2 and the inhibition of KEAP1 via the Akt pathway, as confirmed by specific inhibitors and RNA interference techniques. Moreover, desoxyrhapontigenin ameliorated endotoxin-induced lung inflammation by the up-regulation of HO-1 and Nrf2 and the simultaneous inhibition of p65 expression. Taken together, desoxyrhapontigenin is a new candidate anti-inflammatory or antioxidative agent.
